# Graphene oxide activates canonical TGFβ signalling in a human chondrocyte cell line *via* increased plasma membrane tension†

**DOI:** 10.1039/d3nr06033k

**Published:** 2024-02-19

**Authors:** Leona Ogene, Steven Woods, Joseph Hetmanski, Neus Lozano, Angeliki Karakasidi, Patrick T. Caswell, Kostas Kostarelos, Marco A. N. Domingos, Sandra Vranic, Susan J. Kimber

**Affiliations:** a Division of Cell Matrix Biology & Regenerative Medicine, Faculty of Biology, Medicine and Health, The University of Manchester Manchester M13 9PT UK sue.kimber@manchester.ac.uk +44 (0)161 275 6773; b Wellcome Trust Centre for Cell-Matrix Research, University of Manchester, Manchester Academic Health Science Centre Manchester M13 9PT UK; c Nanomedicine Lab, Catalan Institute of Nanoscience and Nanotechnology (ICN2), CSIC and BIST Campus UAB Bellaterra 08193 Barcelona Spain; d Nano-Cell Biology Lab, Division of Cell Matrix Biology & Regenerative Medicine, School of Biological Sciences, The University of Manchester Manchester M13 9PT UK; e Institució Catalana de Recerca i Estudis Avançats (ICREA) Pg. Lluís Companys 23 Barcelona Spain; f Centre for Nanotechnology in Medicine, Faculty of Biology Medicine & Health, The University of Manchester Manchester UK; g Department of Solids and Structure, School of Engineering, Faculty of Science and Engineering, Henry Royce Institute, The University of Manchester Manchester UK

## Abstract

Graphene Oxide (GO) has been shown to increase the expression of key cartilage genes and matrix components within 3D scaffolds. Understanding the mechanisms behind the chondroinductive ability of GO is critical for developing articular cartilage regeneration therapies but remains poorly understood. The objectives of this work were to elucidate the effects of GO on the key chondrogenic signalling pathway – TGFβ and identify the mechanism through which signal activation is achieved in human chondrocytes. Activation of canonical signalling was validated through GO-induced SMAD-2 phosphorylation and upregulation of known TGFβ response genes, while the use of a TGFβ signalling reporter assay allowed us to identify the onset of GO-induced signal activation which has not been previously reported. Importantly, we investigate the cell–material interactions and molecular mechanisms behind these effects, establishing a novel link between GO, the plasma membrane and intracellular signalling. By leveraging fluorescent lifetime imaging (FLIM) and a membrane tension probe, we reveal GO-mediated increases in plasma membrane tension, in real-time for the first time. Furthermore, we report the activation of mechanosensory pathways which are known to be regulated by changes in plasma membrane tension and reveal the activation of endogenous latent TGFβ in the presence of GO, providing a mechanism for signal activation. The data presented here are critical to understanding the chondroinductive properties of GO and are important for the implementation of GO in regenerative medicine.

## Abstract 2

This study provides new insights into the molecular mechanisms underlying GO activated TGFβ signalling in chondrogenic cells. Contact with GO increased plasma membrane tension, activated mechanosensory pathways and endogenous TGFβ-1 as a mechanism for TGFβ signal activation.

## Graphical abstract

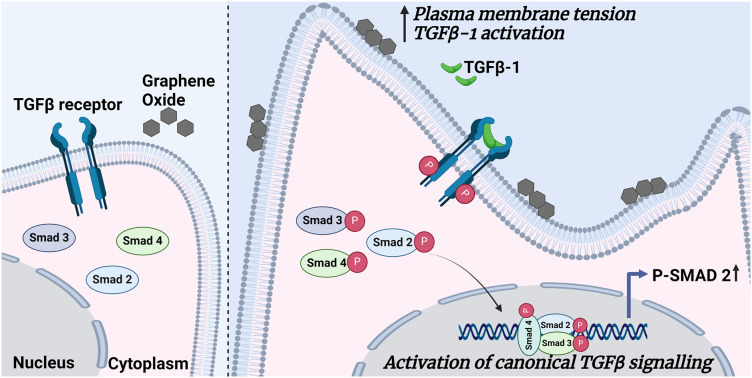

## 1. Introduction

Tissue engineering is an interdisciplinary field which uses principles and methods in biology, chemistry, biomaterial science and engineering, to develop biological substitutes that restore, maintain, or improve functionality of damaged tissues.^1^ As newer and more advanced biomaterials continue to evolve, their macro-level effects on tissue growth and repair are often highlighted. However, detailed research that clarifies cell–material interactions, illuminating the specific cellular mechanisms and molecular pathways behind these responses is less prevalent. Failure to provide in-depth biological analysis can hinder the understanding of how these materials interact with cellular systems, which is crucial for optimising their performance *in vivo*.

Articular cartilage (AC) is a highly specialised type of hyaline cartilage that covers the surfaces of synovial joints, whose primary function is to provide a smooth, low-friction surface for pain-free joint movement.^2^ Trauma, aging, genetics and degenerative diseases such as osteoarthritis can all stimulate the progressive and irreversible break down of articular cartilage.^3^ But the current clinically available therapies for cartilage repair are unable to generate long lasting functional tissues.^4^

Cartilage tissue engineering aims to produce functional alternatives to native cartilage by encapsulating cells within biomaterial scaffolds which promote chondrogenic differentiation. Recent years have seen a surge in the number of studies which utilise Graphene Oxide (GO) for cartilage regeneration.^5–8^ Favourable properties include GO's exceptional mechanical strength,^9^ which can be used to enhance the mechanical properties of scaffolds and improve chondrocyte proliferation and matrix synthesis.^10^ Additionally, GO's exceptionally high surface area to volume ratio and high protein affinity are often utilised to achieve the localised delivery of bioactive molecules that stimulate cell growth and chondrogenic differentiation.^6,11–13^ Zhou *et al.* achieved over 99% efficiency when absorbing 0.6 μg TGFβ-3 onto GO dispersions, before encapsulating them within collagen-based scaffolds to drive MSC-chondrogenesis.^11^

Remarkably, even in the absence of growth factors, GO has been reported to enhance chondrogenic differentiation in adult stem cells, highlighting its potential as a chondroinductive material.^7,11,14^ The use of GO as a substitute for chondrogenic growth factor supplementation could provide a low cost and easily producible method for induction of chondrogenic differentiation with many GOs now commercially available. However, a deeper understanding of the cellular mechanisms behind GO's chondrogenic effects is necessary for successful biomedical applications.

Transforming Growth Factor-βs (TGFβs) are a family of polypeptide growth factors that are essential for driving chondrogenesis and the maintenance of articular chondrocytes.^15^ Canonical TGFβ signalling is propagated through activation of receptors, triggering phosphorylation of the TGFβ specific transcription factors Suppressor of Mothers against Decapentaplegic (SMAD) 2 and 3.^16^ Phosphorylated SMAD2/3 associates with SMAD4 to form an activated complex that enters the nucleus and regulates the transcription of TGFβ-regulated genes. This contrasts to non-canonical TGFβ signalling which involves the activation of pathways independent of SMAD proteins, such as mitogen-activated protein kinase (MAPK) and phosphatidylinositol-3-kinase (PI3K)/AKT signalling.^17^ Therefore, we hypothesise that GO could activate TGFβ signalling in human chondrocytes, a possible mechanism underlying the chondroinductive properties of GO.

To test this hypothesis, we investigated the influence of GO on TGFβ signalling using the human chondrocyte cell line TC28a2. TC28a2 cells have been frequently used as an effective model for primary articular chondrocytes which are limited by dedifferentiation in culture and the low number of chondrocytes extracted per sample.^18–20^ In contrast to previous studies, cells were tested in both a serum- and growth factor-free environment, to assess the direct effects of GO on chondrogenic behaviour and signalling pathways.

Furthermore, we examine the immediate cell–material interactions and molecular pathways that lead to TGFβ signal activation. By elucidating the mechanisms through which GO can modulate the signalling pathways that regulate chondrogenic differentiation, our findings are critical to understanding the use of GO in cartilage tissue engineering applications.

## 2. Materials and methods

### 2.1. Synthesis and characterization of graphene oxide materials

**Fig. 1:**
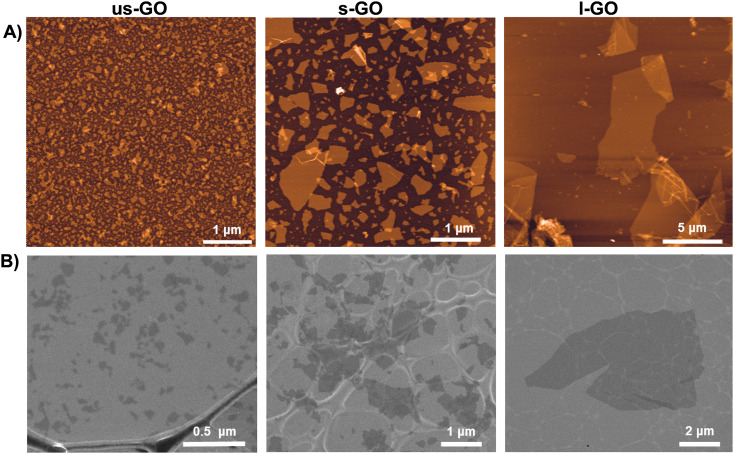
Morphological characterization of us-GO, s-GO and l-GO. (A) Height atomic force microscopy (AFM) images. (B) Scanning electron microscopy (SEM) images.

Biological-grade GO solutions were synthesised in house under endotoxin-free conditions as previously described.^21^ Briefly, ultrasmall (us-GO), small (s-GO), and large GO (l-GO) sheets, only differing in the lateral dimensions (Fig. 1), were produced from graphite powder (Sigma Aldrich, UK) using the modified Hummers’ method (see also Table S1† for thorough physicochemical characterisation of the GO materials used). All three GO materials were prepared in water for injection. Lateral dimensions were measured using Atomic force microscopy (AFM) and Scanning Electron Microscopy (SEM). Further detailed information for the GO characterization can be found in our previous publications.^22,23^

### 2.2. TC28a2 cell culture

Chondrogenic cell line (TC28a2)^24^ was cultured at 37 °C, 5% CO_2_, in 75 cm^2^ cell culture flasks (Corning) with DMEM (Gibco, #11960044) containing 10% w/v foetal bovine serum (Merck, #12103C), 1% w/v l-glutamine (Gibco, #25030081) and 1% w/v penicillin/streptomycin (Gibco, #15140122). For routine maintenance, cells were subcultured into flasks containing fresh warmed medium at a passage ratio of 1 : 10. Briefly, cells were washed with phosphate buffered saline (PBS; Merck, #D8537) before cell dissociation with 5 mL of TryPLE Express solution (Gibco, #12604021). Cells were then centrifuged at 600*g* for 3 min before pellet resuspension in medium and continued culture.

### 2.3. TGFβ signalling reporter assay

TC28a2 cells transfected with luciferase reporter plasmids, SMAD Binding Element nanoluciferase-pest (SBE nLUCp), in which luciferase expression is under control of the SMAD binding element (SBE)^25^ were used to evaluate SMAD-induced transcription and signalling. A total of 10,000 reporter cells were seeded into each well of a black walled 96 well plate and incubated overnight, following which cells were serum starved for a further 24 h. Various concentrations of GO were applied to cells by diluting in serum-free medium, with TGFβ-3 (10 ng ml^−1^) used as a positive control. After 1–24 h Nanoglo live reagent (Promega) was applied to cells and luminescence was read using the GloMax-Multi_ Detection system (Promega). Relative luminesce units (RLU) are a measure of SMAD 2/3 binding (SBE nLUCp activity) reflecting canonical TGF-β signalling.

### 2.4. Assessment of cell viability using the Trypan blue cell viability assay

Cell viability was quantified using the Trypan Blue cell viability assay to determine the mean number of viable cells. TC28a2 cells were seeded in 12 well plates at a density of 0.1 × 10^6^ cells per well and incubated overnight, following which the medium was replaced with serum free medium and cells were serum starved for a further 24 hours. GO was then diluted in serum free medium and applied to cells for 24 hours, then subsequently detached using Trypsin and transferred to an Eppendorf tube and mixed with Trypan blue using a 1 : 1 ratio. The number of live cells was then counted using a haemocytometer.

### 2.5. RNA extraction and qRT-PCR analysis

TC28a2 cells were seeded into 6 well plates at a density of 0.2 × 10^6^ cells per well and incubated overnight, followed by serum starving overnight. Then, cells were treated with 5 μg ml^−1^ of us-GO or with 10 ng ml^−1^ of TGFβ-3 used as a positive control. After 24 hours cells were washed with PBS and detached using TrypLE before centrifuging to form a cell pellet. After centrifugation, the supernatant was discarded, and cells were resuspended in 350 μl of lysis buffer. Next, total RNA was extracted using Monarch® Total RNA Miniprep Kit (New England Biolabs), according to the manufacturer's instructions. The concentration of total RNA was determined using the Nanodrop 2000 and the purity was checked measuring both absorbance ratios 260 nm/280 nm and 260 nm/230 nm, with expected values between 1.8 and 2.0 indicating high RNA purity. RNA was reverse transcribed using the High-Capacity RNA-to-cDNA™ Kit. The qPCR reaction was then prepared using PowerUp SYBR green master mix (ThermoFisher Scientific, #A25742) with 5 ng of cDNA per reaction and 400 nM final concentration forward and reverse primers (Table S2†). The qPCR reaction was run using a BioRad C1000Touch Thermal Cycler using the following cycling conditions: denaturation at 95 °C for 10 min, 39 cycles of 95 °C for 30 s, 60 °C for 30 s and 72 °C for 35 s, final extension at 72 °C for 10 min, and melt curve analysis at 65 °C for 5 s and 95 °C for 30 s. All samples were run in triplicate, and the mean value of each triplicate was used to determine relative gene expression (normalised to GAPDH using the 2^−ΔCT^ method).

### 2.6. Western blotting

TC28a2 cells were seeded into 6-well plates at a density of 0.3 × 10^6^ cells per well before 24 h serum starvation and subsequent exposure to 5 μg ml^−1^ us-GO for 4 hours. Protein was harvested on ice by incubating cells in cell lysis buffer for 30 minutes at 4 °C, followed by centrifugation at 14 000*g* at 4 °C for 10 min. Protein concentration was determined using a Pierce BCA assay kit (Thermo Fisher Scientific, #23225) and diluted to 30–40 μg before boiling at 95 °C for 10 min in lane marker reducing buffer (Thermo Fisher Scientific, #39000). Protein samples were subject to gel electrophoresis at 60 V for 10 minutes followed by 120 V for 1 hour on a BOLT 10% Bis-Tris gel (Thermo Fisher Scientific, #NW00100BOX) with broad range markers (11–245 kDa, NEB P7712S). Protein was transferred using the iBlot-2 Gel Transfer device (Thermo IB21001), using iBlot-2 Transfer stacks (PVDF membrane, Thermo IB23001).

After electro transfer membranes were blocked overnight at 4 °C with 5% BSA in 1× TBS-0.1%Tween-20, then incubated with the following primary antibodies and left overnight on a rocker at 4 °C; P-SMAD2 (1 : 1000 dilution; #3108, Cell Signalling Technologies), SMAD 2 (1 : 1000 dilution, #5339, Cell Signalling Technologies) P-P38 (1 : 2000 dilution, #9216, Cell Signalling Technologies), P38 (1 : 1000 dilution, #9212, Cell Signalling Technologies), P-SAPK/JNK (1 : 1000 dilution, #4668 Cell Signalling Technologies), SAPK/JNK (1 : 1000 dilution, #9252, Cell Signalling Technologies), TGF-β (1 : 500 dilution, #3711, Cell Signalling Technologies) GAPDH (1 : 1000 dilution, #5174, Cell Signalling Technologies) and β-Actin HRP Conjugate (1 : 1000 dilution, #12262, Cell Signalling Technology). Membranes were washed three times with TBS-0.1%Tween-20, then secondary HRP conjugated antibodies; (anti-rabbit IgG, HRP-linked antibody, 1 : 1000 dilution, #7074, Cell Signalling Technology) (anti-mouse IgG, HRP-linked antibody, 1 : 1000 dilution, #7076, Cell Signalling Technology) were added in 5% BSA in TBS-0.1%Tween-20 for 1 hour. After three washes in TBS-0.1%Tween-20, staining was imaged and analysed using the Image Lab software.

### 2.7. Confocal microscopy and plasma membrane staining

To investigate interactions of GO materials with cells, imaging of label-free GO was carried out using the method previously described by Vranic *et al.*^26^ Cells were seeded in a Cellview cell culture dish (627870, Greiner Bio-One Ltd, UK) at a density of 0.5 × 10^5^ cells per well and incubated overnight. Subsequently, cells were serum starved for a further 24 hours before addition of 10 μg ml^−1^ of us-GO in serum-free conditions for 4 or 24 hours. This higher concentration was used to improve detection of us-GO. After this, CellMask green plasma membrane stain (#C37608, Thermo Scientific, UK) was diluted in medium (1 : 2500) and added to the cells. Live cells were examined under a Zeiss 780 confocal laser scanning microscope using a 40× objective. The auto fluorescent properties of GO which enabled label free confocal imaging have been previously described.^26^ The excitation wavelengths used for the CellMask green plasma membrane stain and GOs were 488 nm and 594 nm, respectively. The CellMask green plasma membrane stain had an emission maximum at 520 nm, while the emission wavelength for GOs ranged from 620 nm to 690 nm. Cell imaging was performed at three positions per well, selected without preference, using zoom levels of 0.6× and 1.6× for each position. The acquired images were processed using Zen Black software.

### 2.8. Plasma membrane tension measurements by fluorescent lifetime imaging microscopy (FLIM)

Cells were seeded into culture dishes (Mattek, part no P35G-1.5-10-C) at a density of 0.3 × 10^6^ cells per dish and incubated overnight at 37 °C. Following this, the medium was replaced with 1.8 ml serum-free medium and incubated for a further 24 hours. Stock concentrations of us-GO (50 μg ml^−1^) and TGFβ-3 (100 ng ml^−1^) were prepared and added to cells using a 1 : 10 dilution to give the final conditions of untreated, us-GO (5 μg ml^−1^) or TGFβ-3 (10 ng ml^−1^). After four hours Flipper-TR probe (Spirochrome, cat#: SC020) was added to cells (1 : 1000 dilution) and incubated at 37 °C for 30 minutes before imaging.

Images were collected on a Leica TCS SP8 AOBS inverted gSTED microscope using a 40×/1.30 HC PL APO (Oil) objective and 1× confocal zoom. The confocal settings were as follows, pinhole 1 airy unit, scan speed 50 Hz unidirectional, format 512 × 512. FLIM images were collected using a hybrid detector with 488 nm white-light laser excitation and 575–625 emission as per Flipper-TR recommendations.^27^ Three images were taken per condition and the experiment was repeated *N* = 3 times.

Images were processed using LASX FALCON FLIM software, to determine the lifetime of manually identified membranes pixels in each image, ten regions of interest (ROIs) were randomly selected per image and the lifetime was calculated using a single exponential fit model.

Cells treated with the materials, but unstained with the Flipper probe were also run to ensure that the inherent fluorescence of GOs did not affect the lifetime. A detailed description of the mechanism behind the probe and use with FLIM has been previously reported.^28^

### 2.9. Hydroethidine (HE) oxidation

For detection of intracellular reactive oxygen species (ROS), cells were seeded and treated in triplicate in 12-well plates at a density of 0.2 × 10^6^ cells per well, following which cells were serum starved for 24 hours. One ml solutions of 50 μg ml^−1^ of us-GO, and 10 mM H_2_O_2_ were prepared and added to wells using a 1 in 10 dilution to give a final concentration of 5 μg ml^−1^ us-GO, 1 mM H_2_O_2_ and 400 μM H_2_O_2_, and incubated for 4 hours. After treatment, supernatants were aspirated and cells gently washed once with 1 mL per well of prewarmed PBS (with Ca^2+^/Mg^2+^ Sigma-Aldrich, Merck Sigma, UK). Cells were detached using 0.05% Trypsin–EDTA solution (Sigma-Aldrich, UK) for 5 min, then centrifuged for 5 min at 1500 rpm; supernatants were then aspirated, and pellets containing cells were resuspended in 1 μM hydroethidine (Sigma-Aldrich, Merck Sigma, UK) for 20 min on ice. Ten thousand cells were analysed on a BD FACSVerse flow cytometer using 488 nm excitation and 620 nm band-pass filters for HE detection. Cells treated with GO, but unstained with HE, were also run in order to ensure that the detected signal was not due to the inherent fluorescence of GO.

### 2.10. YAP/TAZ_TEAD activity reporter assay

The TC28a2 Yes-associated protein and TAZ transcriptional coactivator with PDZ-binding motif (YAP/TAZ)_TEAD reporter cell line^29^ was used as a reporter for YAP/TAZ activity and extracellular matrix (ECM) stiffness.

Rho/Rho-associated protein kinase (ROCK) signalling pathways participate in stiffness sensing through stress fibre formation.^29^ ROCK inhibitors have been shown to decrease cell tension.^30^ Therefore, Revitacell (#A2644501, ThermoFisher) containing a specific ROCK inhibitor complex different from Y-27632 was used as a positive control in this experiment.

A total of 10,000 reporter cells were seeded into each well of a black walled 96 well plate and incubated overnight, following which cells were serum starved for a further 24 hours. Then us-GO (5 μg ml^−1^), Revitacell (#A2644501, Gibco) (1 : 100), TGFβ-3 (10 ng ml^−1^) was applied to cells by diluting in serum-free medium. After 4 hours Nanoglo live reagent (Promega) was applied to cells and luminescence was read using the GloMax-Multi Detection system (Promega). The assay output, relative luminesce units (RLU) are a measure of YAP/TAZ activity through interaction with TEAD1.

### 2.11. Statistical analysis

All data was expressed as mean ± standard error of the mean (SEM). Statistical analysis was performed using one-way ANOVA using graph pad prism 9.3.1 with Dunnett multiple comparison test. A *p* value <0.05 was considered as statistically significant.

## 3. Results

### 3.1. Graphene oxide synthesis and characterization

For the specific GO batch used in this work, GO sheets with three different controlled lateral dimensions, with the same physicochemical properties such as thickness or proportion of functionalization, were synthesized under endotoxin-free conditions.^21^ The lateral dimension of the GO flakes measured by AFM and SEM (Fig. 1) were 10 nm–600 nm (95% < 200 nm) for us-GO, 25 nm–2 μm (95% < 1 μm) for s-GO, and 2 μm–20 μm (95% < 14 μm) for l-GO.

### 3.2. Graphene oxide activates TGFβ signalling in TC28a2 human chondrocytes

**Fig. 2:**
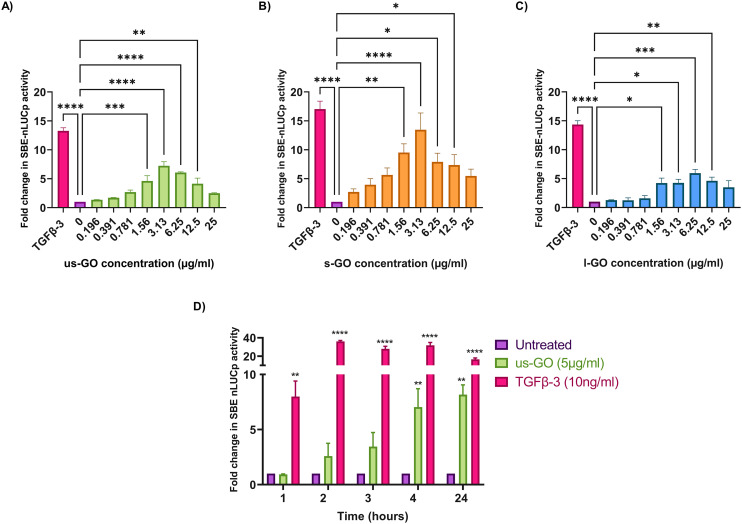
Analysis of SMAD binding element (SBE) induction 24 hours after stimulation with (A) us-GO (B) s-GO and (C) l-GO using a 1 in 2 serial dilution from 25 μg ml^−1^ of GO flakes compared to TGFβ-3 (10 ng ml^−1^) which was used as positive control. (D) Analysis of SBE induction for up to 24 hours after exposure to 5 μg ml^−1^ us-GO or 10 ng ml^−1^ TGFβ-3. Data is presented as mean fold change in NanoLuc-pest luciferase activity (RLU) in comparison to the control (untreated) and bars represent mean ± SEM. *P* values were calculated an ordinary one-way ANOVA (*p < 0.05, **p < 0.01, ***p < 0.005, ****p < 0.0001).

To investigate the effects of GO on TGFβ signalling, TC28a2 with a TGFβ-3 signalling reporter (SBE-nLUCp) were used to monitor SMAD2/3 kinetics (Fig. 2A–D). Signalling activity varied according to size (lateral dimension) of the GO flakes and concentrations used. As expected, TGFβ-3 (positive control) had the most pronounced effects on SMAD signalling activity in TC28a2 cells with an induction of SBE-nLUCp activity of up to 17-fold (*p* < 0.005) compared to untreated cells (*p* < 0.005) at 24 hours.

Signalling activity peaked at 3.13 (μg ml^−1^) in us-GO and s-GO treated samples, with a 7.2-fold and 13.4-fold increase in signalling activity. However, l-GO showed slightly reduced activity, peaking at a higher concentration of 6.25 (μg ml^−1^). The effect of GO in the reporter assay decreased at higher concentrations (Fig. 2A–C).

In contrast to the effect on the TGFβ-SBE reporter, us-GO did not significantly activate the BMP response element (BRE) reporter (Fig. S1†), suggesting GO specifically activates SMAD2/3 TGFβ signalling and did not regulate the parallel SMAD 1/5/8 BMP signalling pathway.

Studies were conducted in serum-starved cells, using serum-free conditions to minimise the interfering effects of serum components on cell signalling pathways. Therefore, cell viability was assessed using Trypan blue to determine that GO did not have deleterious effects on the cells at the concentrations used in serum-free medium (Fig. S2A†). Cells treated with GO in serum free medium displayed dose and size dependent effects of GO on cell viability. L-GO appeared to induce the greatest effect with significant reductions in viability at both 5 μg ml^−1^ (*p* < 0.05) and 25 μg ml^−1^ (*p* < 0.0001) indicating that larger flakes were more toxic to cells. Cytotoxicity with us-GO and s-GO was dependent on GO concentration, and the number of viable cells was not significantly affected by treatment with 5 μg ml^−1^ however, all sizes of GO induced significant reduction in cell viability when cells were treated with 25 μg ml^−1^ of GO after prolonged serum starvation.

Since all GO flakes were able to activate TGFβ signalling, further investigations were carried out with us-GO (5 μg ml^−1^) which had no effect on cell viability (Fig. S2A†). Fig. 2D shows the time course analysis for TC28a2 cells treated with us-GO (5 μg ml^−1^) or TGFβ-3 (10 ng ml^−1^) in comparison to untreated cells at 1, 2, 3, 4 and 24 hours. Activation of canonical signalling by us-GO was delayed in comparison to cells treated with TGFβ-3. Cells treated with the latter showed an approximately 8-fold increase (*p* < 0.001) in canonical signalling activity (*p* < 0.01) at 1 hour. However significant SMAD2/3 TGFβ signalling activity was not observed until 4 hours with us-GO.

**Fig. 3:**
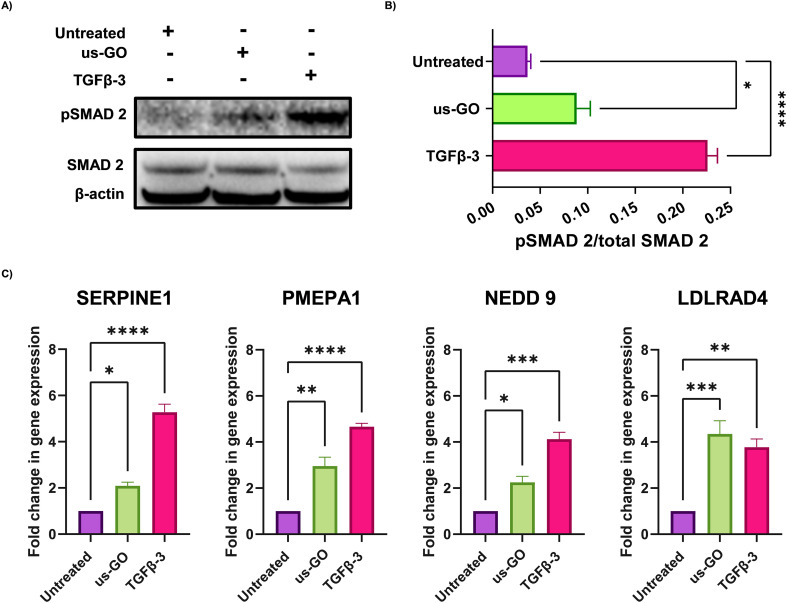
Ultrasmall graphene oxide (us-GO) activates canonical TGFβ signalling in TC28a2 cells. (A) Western blot analysis of the protein expression of PSMAD-2 and SMAD 2 in TC28a2 cells 4 hours after treatment with us-GO (5 μg ml^−1^) or TGFβ-3 (10 ng ml^−1^). Quantification of P-SMAD 2/SMAD 2 protein expression levels normalised to Beta actin. (B) Analysis of the expression of TGFβ response genes in TC28a2 cells 24 hours after treatment with us-GO. Gene expression was normalised to GAPDH. Results are presented as fold increase in gene expression normalised to untreated cells. Bars represent mean ± SEM, *N* ≥ 3. *P* values were calculated using an ordinary one-way ANOVA (*p < 0.05, **p < 0.01, ***p < 0.005, ****p < 0.0001).

These findings were supported by qualitative and quantitative (Fig. 3A) analysis of pSMAD 2 immunoblots which showed a significant increase in SMAD 2 phosphorylation after treatment with us-GO, indicative of canonical TGFβ signalling.

For further validation of us-GO activated TGFβ signalling TC28a2, cells were stimulated with us-GO (5 μg ml^−1^) or TGFβ-3 (10 ng ml^−1^) for 24 hours before harvesting and extracting RNA to analyse the expression of known TC28a2 TGFβ response genes (ArrayExpress E-MTAB-10279 Woods *et al.*^25^) (Table S3†). RT-qPCR analysis showed that both treatment with us-GO and TGFβ-3 significantly increased gene expression of SERPINE 1, PMEPA1, NEDD 9 and LDLRD4 (Fig. 3C).

TGFβ signalling may also occur through a non-canonical signalling pathway involving p38 and JNK which can be activated by the presence of reactive oxygen species (ROS).^31^ Therefore, to determine whether GO activation also occurred through non-canonical pathways, we first assessed intracellular ROS production using flow cytometry with the HE probe (Fig. S3A†). Treatment with us-GO induced a significant increase in ROS levels after four hours compared to untreated cells, however this did not affect cell viability (Fig. S3B†). Additionally, negligible effects of us-GO on p38 or JNK phosphorylation were observed after 4 hours (Fig. S3D and E†). This suggests activation of TGFβ signalling does not occur through the TAK1-JNK/p38 non canonical TGFβ signalling pathway.

### 3.3. Ultrasmall graphene oxide is strongly attached to the plasma membrane of TC28a2 cells

**Fig. 4:**
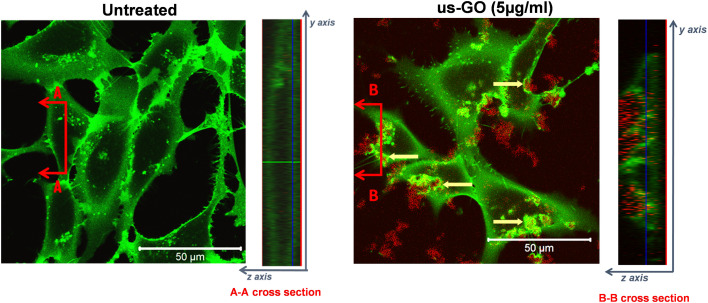
Interaction of us-GO (10 μg ml^−1^) with the plasma membrane of TC28a2 cells labelled with CellMask (green) in serum free medium after 24 hours in comparison to untreated cells. Images were taken using a Zeiss 780 multiphoton confocal laser scanning microscope and a 40× objective. Cross sections (right) were used to determine whether GO was interacting inside or outside of the cell. Yellow arrows indicate regions of combined CellMask (green) and us-GO (red) fluorescence where the plasma membrane was interacting with GO.

To further our understanding of the mechanism by which GO activates TGFβ signalling, we used confocal microscopy to determine whether us-GO was interacting with cells at the plasma membrane or being taken up by the cells (Fig. 4). The cross section denoted B–B depicts membrane ruffling and the accumulation of us-GO (red) mainly on the top of the plasma membrane. However, no uptake of us-GO by TC28a2 cells was observed after 24 hours.

We also acquired representative phase microscopy images, which show the gradual accumulation of GO on the plasma membrane after 1, 4 and 24 hours (Fig. S4†). At 1 hour GO flakes were difficult to observe due to excellent dispersion in the culture medium, however by 4 hours agglomeration and close association of GO to the plasma membrane was visible and remained bound after multiple PBS washes.

### 3.4. Ultrasmall graphene oxide increases plasma membrane tension

**Fig. 5:**
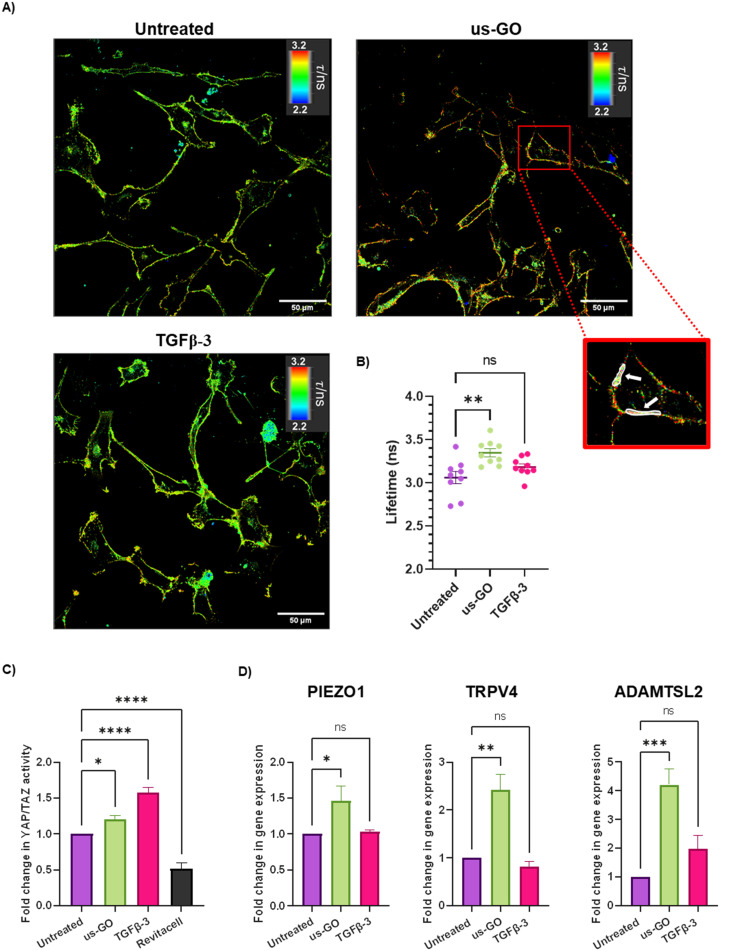
Ultrasmall graphene oxide (us-GO) increases plasma membrane stiffness and activates mechanosensory pathways (A) Fluorescent lifetime imaging (FLIM) analysis of plasma membrane stiffness in untreated, us-GO (5 μg ml^−1^) treated- and TGFβ-3 (10 ng ml^−1^) treated-cells after 4 hours. Images were taken using a Leica SP8 Inverted 3D FLIM confocal microscope using a 40× objective and analysed using LASX FLIM software version 3.56. Scale bar represents 50 μm. White arrows indicate examples of ROIs used to calculate lifetimes. (B) Quantification of lifetime analysis. Each data point represents lifetime (ns) generated from 10 ROIs per image. Values in us-GO group were subtracted by measuring (negligible) background lifetime (0.048 ns) from GO autofluorescence. (C) Analysis of active YAP/TAZ activity *via* TEAD induction 4 hours after stimulation with us-GO (5 μg ml^−1^) 10 ng ml^−1^ TGFβ- or Revitacell which was used as a positivecontrol. (D) Gene expression analysis of PIEZO1, TRPV4 and ADAMTSL2 24 hours after treatment with us-GO. Gene expression was normalised to GAPDH. Results are presented as fold increase in gene expression normalised to untreated cells. Data is presented as mean ± SEM, *P* values were calculated using an ordinary one-way ANOVA (*p < 0.05, **p < 0.01, ***p < 0.005, ****p < 0.0001).

After observing that GO remained attached to the plasma membrane after multiple washes, Flipper-TR plasma membrane tension probe was used to determine whether the attachment of us-GO to TC28a2 cells induced any mechanical changes in the plasma membrane. Cells were stained with Flipper-TR, a fluorescent probe that specifically targets the plasma membrane of cells and reports changes in plasma membrane tension through changes in fluorescent lifetime.^28^ In this assay a higher lifetime is indicative of higher plasma membrane tension. FLIM analysis demonstrated increase in plasma membrane tension in cells treated with us-GO (Fig. 5A and B). Cells treated with us-GO but not the Flipper-TR probe had negligible lifetime values suggesting us-GO auto-fluorescence did not influence the lifetime results (Fig. S5†).

### 3.5. Ultrasmall graphene oxide activation of mechanosensory pathways

The Hippo pathway including YAP/TAZ, and mechanosensitive ion channels are widely studied mechanosensors which sense and respond to changes in mechanical forces exerted on cells.^32,33^ The Hippo pathway negatively regulates TEAD transcription through phosphorylation of YAP and TAZ, co-activators of mechanosensitive TEAD.^34,35^ We used a YAP/TAZ_/TEAD Luciferase Reporter to investigate changes in this pathway in response to us-GO (Fig. 5C). YAP/TAZ signalling activity was significantly upregulated by both us-GO (*P* < 0.05) and TGFβ-3 (*P* < 0.001) and significantly down regulated by Revitacell (*P* < 0.001) which was used as a positive control (Fig. 5C).

Furthermore, gene expression of mechanosensitive ion channels PIEZO1 and TRPV4 and ADAMTSL2 (Fig. 5D) were also significantly upregulated in response to us-GO, which have previously been linked to membrane tension.^29,32,36,37^

### 3.6. Ultrasmall graphene oxide activates endogenous latent TGFβ

**Fig. 6:**
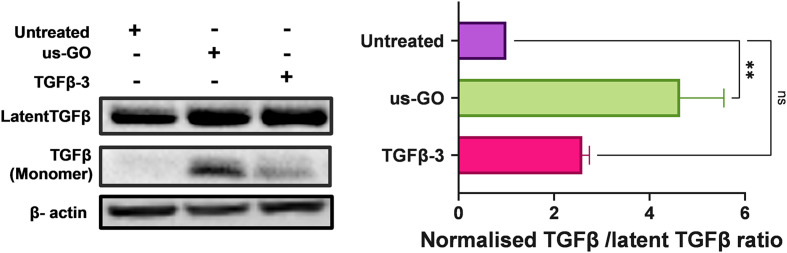
Latent TGFβ-1 is activated in the presence of us-GO. Western blot analysis of the protein expression of endogenous TGFβ-1 in TC28a2 cells 4 hours after treatment with us-GO (5 μg ml^−1^) or TGFβ-3 (10 ng ml^−1^). Quantification of active TGF β-1/and latent TGFβ-1 protein expression levels normalised to Beta actin. Bars represent mean ± SEM, *N* = 3. *P* values were calculated using an ordinary one-way ANOVA (**p < 0.01).

TGFβ can be mechanically activated through cell generated tension which releases active TGFβ from the latent complex to bind receptors and activate signalling.^38^ Therefore, we hypothesised that GO may mechanically activate latent TGFβ through increased plasma membrane tension. To test this hypothesis, we used Western blotting and densitometry analysis to quantify the levels of endogenous active and latent TGFβ-1 in TC28a2 cells after 4 hours of treatment with us-GO or TGFβ-3. While all groups contained high amounts of latent TGFβ, untreated cells showed negligible amounts of active TGFβ monomers (Fig. 6). In support of our hypothesis, the presence of TGFβ monomers was highest in cells treated with us-GO which demonstrated a significant increase (*p* < 0.01) in activation of latent TGFβ. A slight but insignificant increase in active TGFβ was observed in TGFβ-3 treated cells.

## 4. Discussion

### 4.1. Activation of canonical TGFβ signalling by us-GO

Through use of a TGFβ signalling reporter, we demonstrated activation of canonical TGFβ signalling in the TC28a2 human chondrocyte cell line. Activation of TGFβ signalling following interaction with these thin GO nanosheets of a very consistent and reproducible structural and surface character (Table S1†), was further demonstrated *via* P-SMAD 2 western blotting and significant upregulation of several TGFβ response genes using RT-qPCR (Fig. 2). During chondrogenic protocols, activation of the TGFβ signalling pathway typically involves targeting with carefully selected growth factors which bind to specific receptors in the plasma membrane. Yet in our work, simply by addition of the GO nanosheets to serum-free cell culture medium, significant activation of TGFβ was achieved. Furthermore, we provide new data identifying the timing of onset of the GO-TGFβ signalling response at approximately 2–4 hours, and of the activation of endogenous TGFβ-1 by us-GO in TC28a2 cells. These results provide new insights on the mechanisms behind the chondroinductive effects of GO in tissue engineering applications.

Growth factors (such as TGFβs) are essential drivers of chondrogenic differentiation and cartilage homeostasis, however their clinical translatability is limited due to safety and cost effectiveness issues.^39^ Hence, the development of regenerative approaches that reduce or eliminate the need for growth factor supplementation is needed to facilitate safer, less expensive clinical applications of cartilage tissue engineering. In response, recent years have seen the emergence of biomaterial-based strategies to stimulate growth factor signalling. The use of GO as a substitute for chondrogenic growth factors was previously investigated by Shen *et al.* who seeded human bone marrow mesenchymal stem cells (hBMSCs) onto poly-d,l-lactic acid/polyethylene glycol (PDLLA) scaffolds which were then cultured in TGFβ-3 free chondrogenic medium.^14^ After four weeks hBMSCs cultured in GO/PDLLA hydrogels showed significantly higher expression of collagen type II (COL II) and Aggrecan (ACAN) and increased glycosaminoglycan staining compared to those encapsulated in PDLLA scaffolds. Olate-Moya *et al.* also reported enhanced chondrogenesis in growth factor-free conditions after addition of GO. Lower concentrations of GO were able to improve homogenous cell distribution leading to improved matrix deposition.^7^ However, neither study compare the differentiation outcomes of factor-free GO incorporated hydrogels, with hydrogels supplemented with TGFβ. Therefore, it is difficult to determine whether GO incorporation alone is sufficient to produce equivalent robust cartilage tissues to that with TGFβ.

Strategies to achieve chondrogenic differentiation without inducing a hypertrophic phenotype has been the focus of cartilage stem cell differentiation for many years, particularly with mesenchymal stem cell (MSC) based approaches.^40^ Notably, our preliminary data (Fig. S1†) showed treatment with us-GO did not induce BMP signalling in TC28a2 cells, highlighting the specificity of signal activation to the TGFβ signalling arm of the TGFβ superfamily. This specificity is important due to inhibitory or synergistic interactions between TGFβ and other signalling pathways which control chondrogenesis and that can lead to hypertrophic phenotypes. In particular, BMP signalling is known to induce hypertrophy in chondrocytes if not carefully regulated.^40–42^ Nevertheless, while significantly higher than in untreated cells, GO activated TGFβ signalling was still considerably lower than in cells treated with the growth factor. Together these findings point towards the use of GO to enhance the action of growth factors as opposed to replacing them entirely.

In this study we also observed a decrease in TGFβ signalling at higher GO concentrations (Fig. 2A–C), which was likely due to the reduction in cell viability observed in our cytotoxicity studies (Fig. S2A†).^43^ Like others, we observed concentration and size dependent effects on cytotoxicity (Fig. S2†), with larger GO flakes and higher concentrations inducing more cytotoxicity.^26,44^ Possible explanations behind GO induced toxicity may include lacerations to the plasma membrane by larger flakes, and obstruction of oxygen and nutrients exchange at higher concentrations due to absorption onto the plasma membrane. The toxicity induced by us-GO and s-GO in serum free conditions contrasts to previous work.^26^ However in our work cells were exposed to GO in serum free medium after prolonged serum starvation (24 hours) as opposed to only starving cells for sufficient time for GO to interact with the cells (4 hours). Furthermore, in the presence of serum (Fig. S2B†) we observed no cytotoxic effects of us-GO or s-GO. The protective effects of serum and protein coating of GO flakes have been previously discussed.^26,45^

### 4.2. Mechanism of TGF β signalling activation by GO

Only two previous studies have investigated the direct effects of graphene-based materials on TGFβ signalling and TGFβ induced cell responses.^43,46^ Li *et al.* produced evidence of ROS induced MAPK activation as a potential mechanism for Graphene activated TGFβ signalling.^46^ In this study, RAW 264.7 cells treated with 20 μg mL^−1^ of pristine graphene for 48 hours exhibited significantly increased levels of phosphorylated JNK, ERK, p38 and SMAD 2 revealed by Western blotting. Zhu *et al.* reported TGFβ induced epithelial–mesenchymal transition (EMT) in cancer cells after exposure to Graphene Oxide (10 μg ml^−1^) for 24 hours.^43^ Activation of canonical TGFβ signalling *via* increased SMAD2/3 phosphorylation was demonstrated through Western blotting and by increased expression of the TGFβ receptor after 24 hours treatment with GO. However, in our study we investigated the immediate mechanism of signalling activation within chondrogenic cells at 4-hours, which is the time when significant us-GO activated TGFβ signalling was identified through the reporter assay (Fig. 2D). Furthermore, in contrast to the findings of Li *et al.*,^46^ the production of ROS after GO treatment in our study did not coincide with the activation of JNK or p38 (Fig. S3†).

Further investigations were initiated by the observation of robust association of us-GO to the plasma membrane, even after multiple PBS washes (Fig. S4†). Many studies have also reported the attachment of GO to the plasma membrane of mammalian cell lines, resulting in membrane shedding, ruffling, collapse, and fragmentation.^26,47,48^ However, due to the complexity of the plasma membrane, it is difficult to isolate one single interaction as the sole contributing factor to this behaviour. *In vivo*, TGFβ is secreted as a large latent complex which is sequestered into the extracellular matrix. Association of TGFβ dimers with latency associated peptide (LAP) and latent TGFβ binding protein (LTBP) prevents TGFβ from interacting with its receptors and thus renders the growth factor inactive.^49^ Interestingly, evidence of strongly attached GO flakes to the plasma membrane coincided with, increased plasma membrane tension (Fig. 5), activation of endogenous latent TGFβ (Fig. 6) and the onset of activation of TGFβ signalling (Fig. 2D). In addition, ADAMTSL2, an ECM glycoprotein that regulates the availability of active TGFβ dimers by cleaving LTBP,^50^ was also upregulated by us-GO with over a 4 fold increase in gene expression. Altogether, these results provide new insights into a potential molecular mechanism of GO activated TGFβ signalling, whereby the interaction of us-GO with the plasma membrane increases cell tension, thus activating TGFβ from the latent complex.

## 5. Conclusion

Understanding how chondrocytes respond to scaffolds and the biomaterials these scaffolds are made from is critical for tissue engineered AC repair, to provide a viable therapy. Until now, the chondroinductive abilities of GO have largely been attributed to concentration of serum proteins or growth factors which are rapidly absorbed onto the surface of GO.^6,14,51^ However, we have demonstrated enhanced TGFβ signalling in a chondrogenic cell line in the absence of both serum and added growth factors, demonstrating intrinsic, potentially chondroinductive abilities of GO through its ability to activate signalling pathways which control chondrogenesis.

We also demonstrate increased plasma membrane tension, activation of mechanotransduction and activation of latent TGFβ in the presence of us-GO, offering new molecular insights which reinforce previous studies suggesting GO could be used to enhance chondrogenic differentiation. Prior to this study, the effects of GO on plasma membrane tension had not been reported. Furthermore, our work provides a novel method utilising FLIM and a fluorescent probe for characterising the effects of biomaterials on plasma membrane tension in live cells. An advantage of this technique over conventional approaches that employ AFM, is the ease of sample preparation which only requires the addition of the plasma membrane probe to the cell culture medium before imaging as opposed to complex assembly and calibration of a cantilever.

Our future work will focus on investigating whether GO can be used to drive chondrogenesis in pluripotent stem cell derived chondroprogenitors, within photo-cross linkable hydrogel scaffolds.

## Author contributions

Experimental design was created by LO, SW, SV and SJK. LO performed all experiments and data analysis. LO and AK performed HE oxidation assay and data analysis, LO, JH and PC performed FLIM and FLIPPER plasma membrane analysis. NL and KK synthesized all GO materials and provided the material characterization used in this work. The manuscript was written by LO and reviewed and edited by SJK, SV, SW and MD All authors have read and agreed to the published version of the manuscript.

## Data availability

No new data. Data sharing not applicable to this article as no datasets were generated or analysed during the current study.

## Conflicts of interest

The authors declare no conflict of interest.

## Supplementary Material

NR-016-D3NR06033K-s001
