# Supplementary material for: Graphene oxide activates canonical TGFβ signalling in a human chondrocyte cell line via increased plasma membrane tension
Source: Nanoscale. 2024 Feb 19;16(11):5653–64. doi: 10.1039/d3nr06033k (PMC10939054; doi:10.1039/d3nr06033k)
Supplement: NR-016-D3NR06033K-s001 [file NR-016-D3NR06033K-s001.pdf]

Electronic Supplementary Material (ESI) for Nanoscale.
This journal is © The Royal Society of Chemistry 2024

Supplementary

Physicochemical properties	Experimental Technique	us-GO	s-GO	l-GO
Lateral dimension	Optical Microscopy	Non detectable (< 2µm)	Non detectable (< 2µm)	5.0 µm - 25.0 µm (n=82)<br>95% < 23.0 µm<br>Mean 9.8 µm
	Scanning Electron Microscopy (SEM)	10 nm - 310 nm (n=560)<br>95% < 150 nm<br>Mean 77 nm	50 nm - 1.9 µm (n=624)<br>95% < 850 nm<br>Mean 332 nm	5.0 µm - 17.5 µm (n=18)<br>95% < 13.5 µm<br>Mean 10.8 µm
	Atomic Force Microscopy (AFM)	10 nm -590 nm (n=4518)<br>95% < 230nm<br>Mean 47 nm	25 nm - 1.5 µm (n=1426)<br>95% < 475 nm<br>Mean 87 nm	NA (> 20µm)
	Dynamic Light Scattering (DLS)	100.7 ± 1.8 nm<br>PdI: 0.317 ± 0.038	270.9 ± 2.0 nm<br>PdI: 0.298 ± 0.013	2211.0 ± 84.6 nm<br>PdI: 0.977 ± 0.039
Thickness	Atomic Force Microscopy (AFM)	1 - 2 nm	1 - 2 nm	1 - 2 nm
Optical properties	Absorption spectroscopy	ε_{230} (mL µg^{-1} cm^{-1})= 0.048	ε_{230} (mL µg^{-1} cm^{-1})= 0.053	ε_{230} (mL µg^{-1} cm^{-1})= 0.042
Degree of defects (I_D/I_G) $_{633nm}$	Raman spectroscopy (n=5)	1.18 ± 0.02	1.14 ± 0.03	1.27 ± 0.03
Peak (2θ)<br>Interlayer distance (nm)	X-Ray Diffraction (XRD)	11.64 °<br>0.76	12.44 °<br>0.71	12.29 °<br>0.71
Surface charge (ζ-Potential)	Electrophoretic mobility	-56.5 ± 1.3 mV	-52.1 ± 0.4 mV	-46.9 ± 1.0 mV
Chemical composition (%)<br>Purity %(C+O)<br>C:O ratio	X-Ray Photoelectron Spectroscopy (XPS)	C: 71.9 %, O: 24.9 %, S: 0.6 %, B: 2.7 %<br>96.7%<br>2.9	C: 72.2 %, O: 25.0 %, S: 1.2 %, B: 1.6 %<br>97.2%<br>2.9	C: 73.8 %, O: 24.6 %, N: 0.60 %, S: 1.0 %<br>98.4%<br>3.0

n-number in optical microscopy, scanning electron micriscopy and atomic force miscroscopy indicated the number of individual GO sheets analyzed.

Table S1. *Main physicochemical properties of the specific GO nanosheets used in the present study. Properties including lateral dimensions, thickness, optical properties, degree of defects, interlayer distance, surface charge, functionalization degree, chemical composition, and purity, are indicated below.*

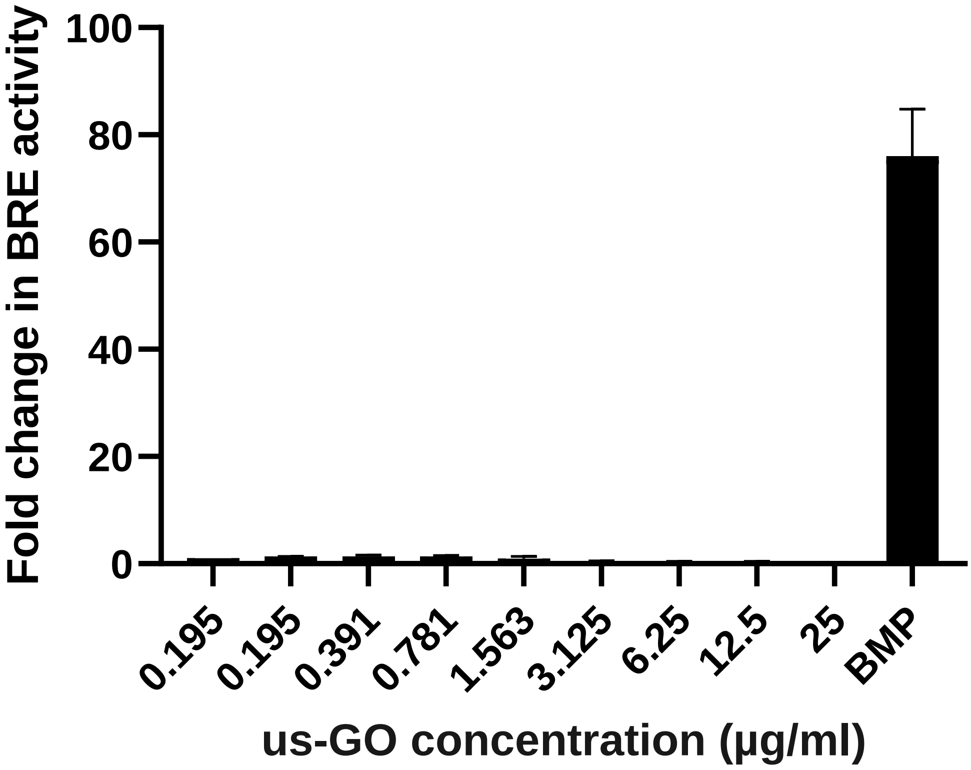

S1. A BMP reporter vector was constructed as previously described, containing the BMP response element (BRE). BRE *activity was measured 24 hours after stimulation with us-GO or BMP-2 (50ng/ml) which was used as a positive control. N=1. The assay output relative luminescent units (RLU)) is a measure of BRE-nLUCp activity (BMP signalling activity).*

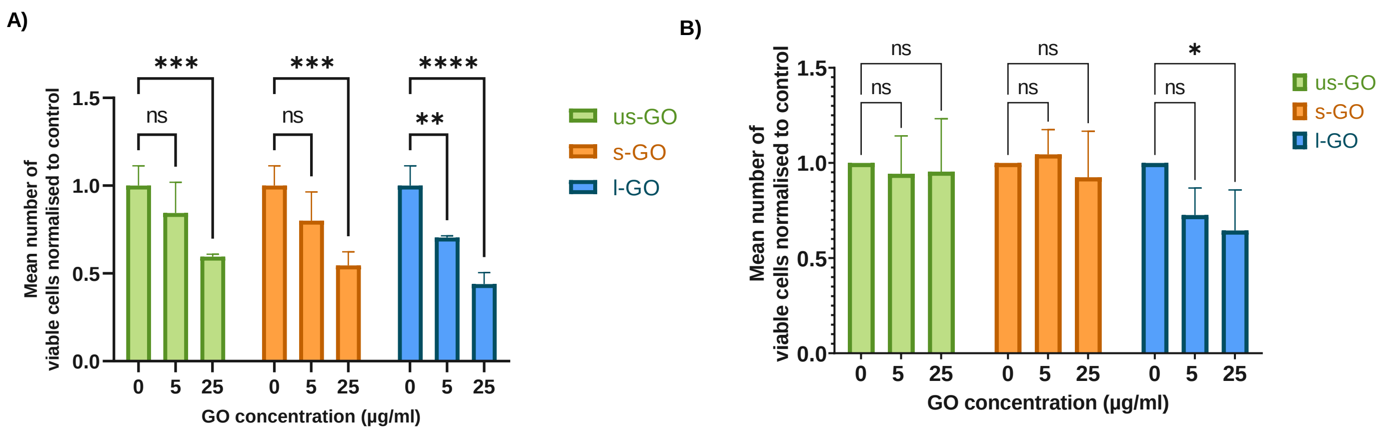

S2.A) Assessment of cell viability in serum starved conditions after 24 hours of serum starvation, followed by a further 24 hours with GO treatment in serum starved conditions. B) Assessment of the cytotoxic effects of us-GO, s-G0 and l-GO on TC28a2 *cells after 24 hours in serum containing media (10% FBS). In both studies mean number of cells was counted using the Trypan blue cell viability assay. Bars represent mean ± SD from three independent studies. P values were calculated using a two- way ordinary one-way ANOVA (*p < 0.05,) on GraphPad Prism 9.3 (*p < 0.05, **p < 0.01, ***p < 0.005, ****p < 0.0001)*

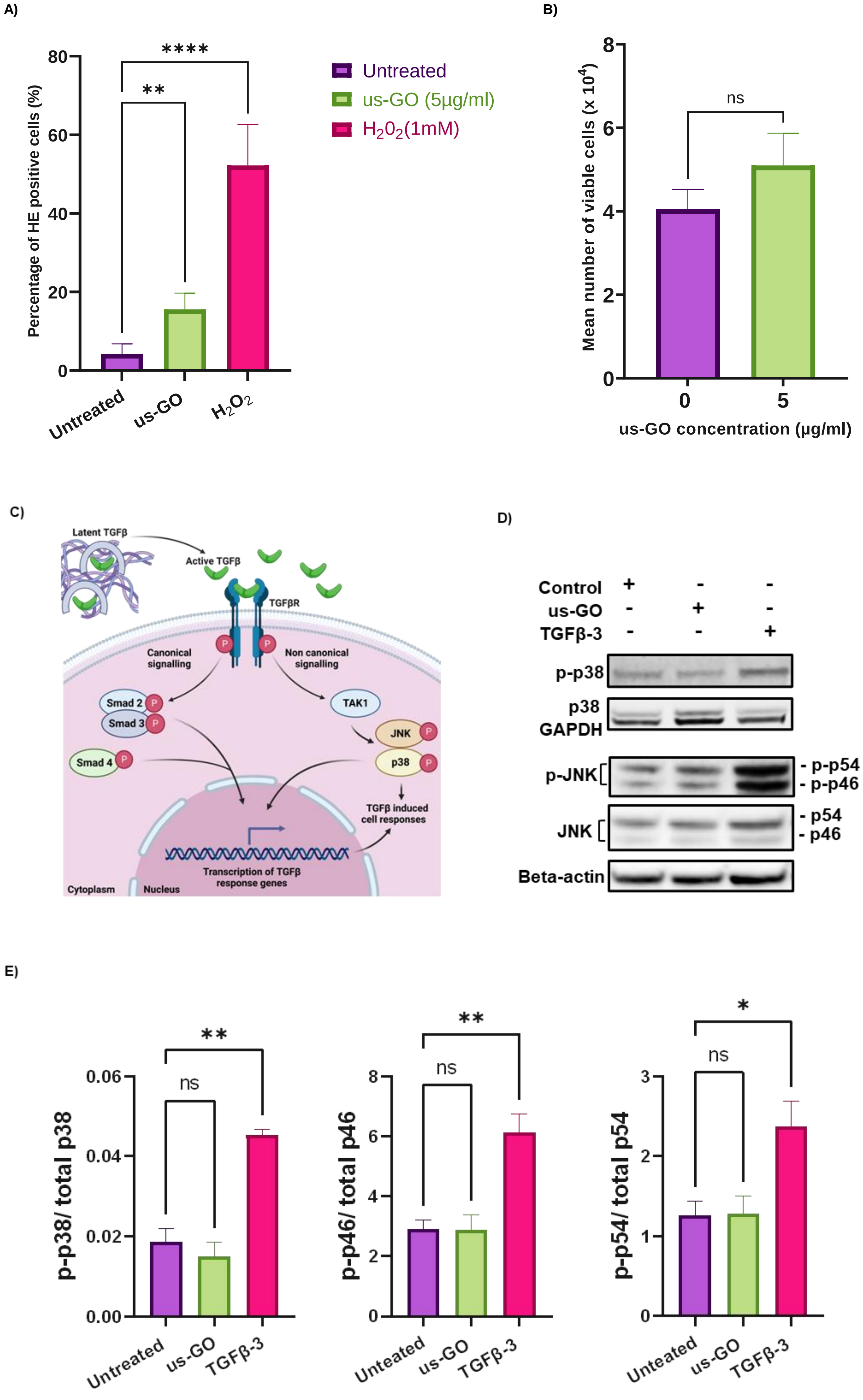

Fig S3. ***Investigating the effects of us-GO on activation of the non canonical TGFβ signalling pathway.*** *A) Study of the intracellular ROS production after treatment with us-GO (5µg/ml) or H_2O_2 (1mM) for 4 h using HE probe. Results are represented as mean % of HE-positive cells. B) The cytotoxic effects of us-GO on TC28a2 cells after 4 hours in serum free media. C) Schematic illustrating activation of canonical and non canonical TGFβ signalling pathways such as the TAK 1 –JNK/p38 pathway. D) Western blot analysis of the phosphorylation of JNK and p38 in TC28a2 cells 4 hours after treatment with us-GO (5µg/ml) or TGFβ-3 (10ng/ml). D) Quantification P-p38/p38, P-p54/p54 and P-p46/p46 protein expression levels normalised to Beta actin or GAPDH .Data is presented as mean ± standard error of the mean (SEM (N=3) P values were calculated using an ordinary one-way ANOVA (*p < 0.05, **p < 0.01, ***p < 0.005, ****p < 0.0001).*

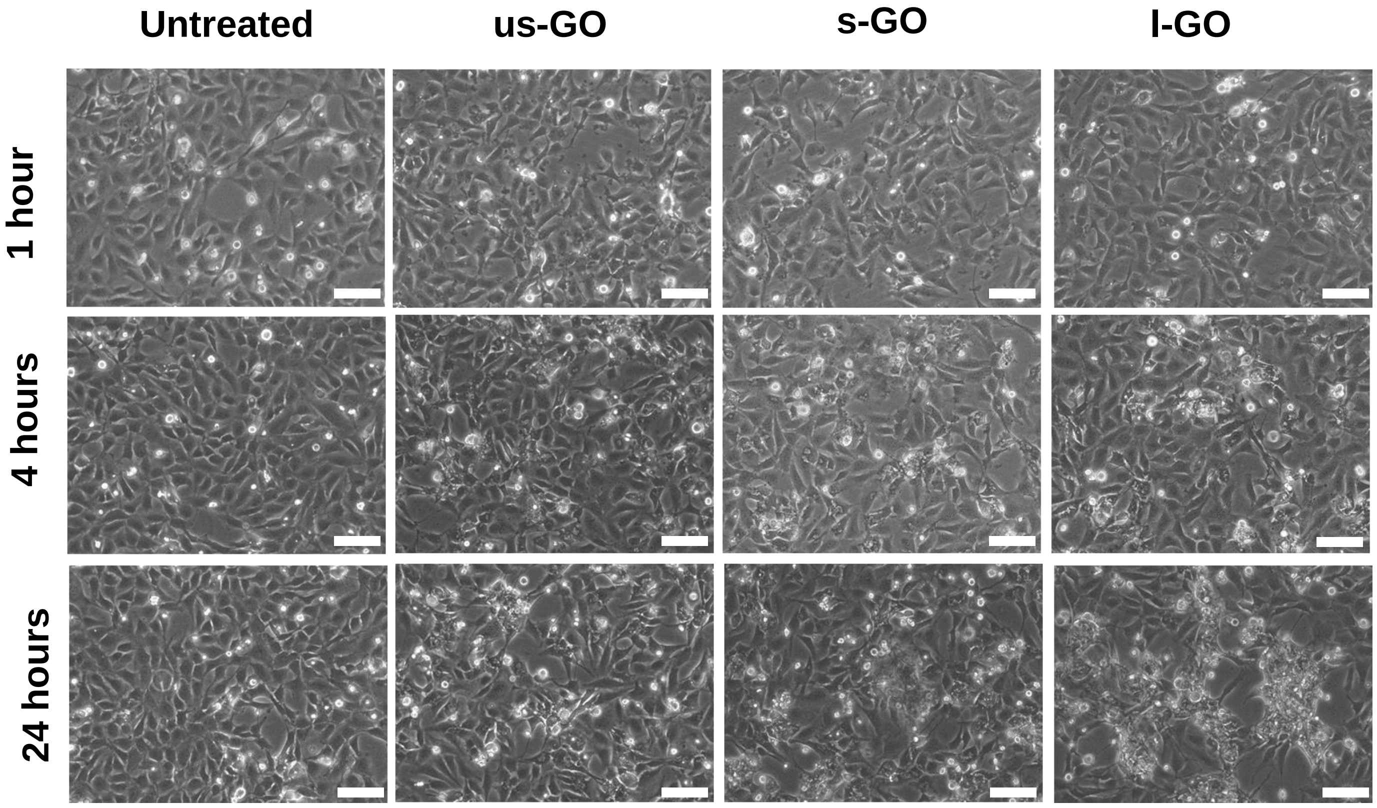

S4. Phase microscopy images of TC28A2 cells after exposure to 5µg/ml of ultrasmall, small or large graphene oxide which show attachment and accumulation of graphene oxide on the plasma membrane from 4 hours, after washing with PBS (3x). Scale bars indicate 100µm

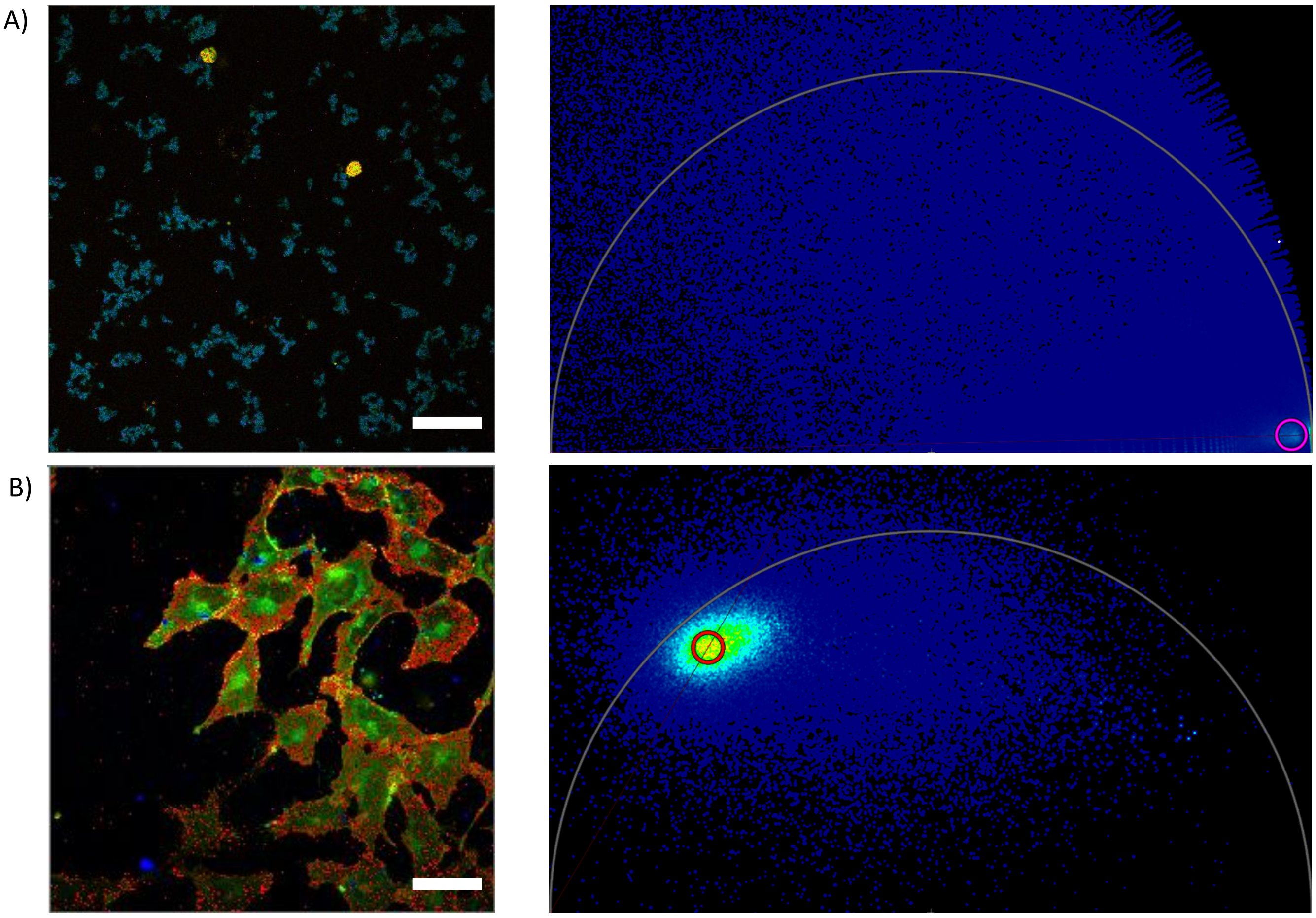

S5) Phasor Fluorescent lifetime imaging (FLIM) colour map of A) TC28a2 cells treated with us-GO (5µg/ml) without addition of FLIPPER probe or B) with addition of FLIPPER probe. Each pixel in the FLIM micrograph corresponds to a point in the phasor plot (right) Lifetime was calculated at maximum population (circled), corresponding pixels are mapped onto the FLIM micrograph (left) demonstrating negligible lifetimes due to GO autofluorescence. Lifetime at circle in A = 0.048, lifetime at circle in B= 3.532. Scale bars indicate 20 microns (N=1).

Gene	Forward primer	Reverse primer
GAPDH	ATGGGGAAGGTGAAGGTCG	TAAAAGCAGCCCTGGTGACC
PMEPA1	CACTACAAGCTGTCTGCACG	ACAGGCATCCTTCTGAGGAC
NEDD 9	CGTCCACCTACAGGGTAAGG	CTGAGAGGGCTTCCACTTCG
SERPINE 1	GACCTCAGGAAGCCCCTAGA	CACCGTGCCACTCTCGTT
LDLRAD4	TTCACCTGCACCAGTGGTAA	GATGATGATTTGGGCGAACT
TRPV4	GCCAGTGTATTCCTCGCTTT	ATGACCTGGCACACAGGTA
PIEZO1	ATCGCCATCATCTGGTTCCC	TGGTGAACAGCGGCTCATAG

Table S2 List of Primer Sequences used for RT-qPCR reactions

		TGFβ-3 vs no growth factor	
Gene name	Mean read count	log2FoldChange	padj
SMAD2	906.9386718	0.101284939	0.53053539
SMAD3	2536.648188	-0.416058566	0.04742297
SMAD4	695.898708	0.307203334	0.1048764
BETA ACTIN	33274.1837	0.362676892	0.04701256
GAPDH	28416.53108	0.138646269	0.58542343
NEDD9	786.6612177	2.640643716	1.09E-41
SERPINE1	19212.75309	3.148428574	9.92E-30
PMEPA1	12284.61143	2.057641851	2.37E-29
LDLRAD4	117.460359	4.902514619	1.74E-25
YAP1	2010.984603	0.189442604	0.30281566
TAZ	98.47911166	-0.478538964	9.65E-02
TEAD 1	2841.684862	0.287407278	0.10029289
PIEZO1	3629.12776	0.65862458	0.01450347
TRPV4	57.46309824	0.21675114	0.64638096
ADAMTSL2	14.22379513	5.638247142	0.00358073

Table S3 RNA seq data extracted from Woods et al (2021) for all relevant transcripts related to experiments.

Antigen	Host	Dilution factor	Molecular weight (kDa)	Company	Catalogue
P SMAD 2	Rabbit	1:1000	60	Cell signalling technology	3108
SMAD 2	Rabbit	1:1000	60	Cell signalling technology	5339
P38 MAPK	Rabbit	1:1000	40	Cell signalling technology	9212
P-P38	Mouse	1:2000	43	Cell signalling technology	9216
Phospho-SAPK/JNK	Rabbit	1:1000	46,54	Cell signalling technology	4668
SAPK/JNK	Rabbit	1:1000	46,54	Cell signalling technology	9252
β-Actin (HRP Conjugate)	Mouse	1:1000	45	Cell signalling technology	12262
TGF-β	Rabbit	1:500	12, 25, 45 to 65	Cell signalling technology	3711
GAPDH	Rabbit	1:1000	37	Cell signalling technology	5174

Table S4 List of Antibody's used for western blotting
